# A Facile Synthesis of 5′-Fluoro-5′-deoxyacadesine (5′-F-AICAR): A Novel Non-phosphorylable AICAR Analogue

**DOI:** 10.3390/molecules171113036

**Published:** 2012-11-02

**Authors:** Stefano D’Errico, Giorgia Oliviero, Nicola Borbone, Jussara Amato, Daniele D’Alonzo, Vincenzo Piccialli, Luciano Mayol, Gennaro Piccialli

**Affiliations:** 1 Dipartimento di Chimica delle Sostanze Naturali, Università degli Studi di Napoli Federico II, Via D. Montesano 49, 80131, Napoli, Italy; 2 Dipartimento di Scienze Chimiche, Università degli Studi di Napoli Federico II, Via Cintia 21, 80126, Napoli, Italy

**Keywords:** AICAR, ZMP, AMPK, AMPK activation, fluorinated nucleosides, fluorination, imidazole nucleosides, nucleoside analogues, modified nucleosides

## Abstract

The substitution of a hydroxyl group by a fluorine atom in a potential drug is an efficient reaction that can, in principle, improve its pharmacological properties. Herein, the synthesis of the novel compound 5′-fluoro-5′-deoxyacadesine (5′-F-AICAR), a strict analogue of AICAR that cannot be 5′-phosphorylated to ZMP by cellular kinases, is reported.

## 1. Introduction

**Figure 1:**
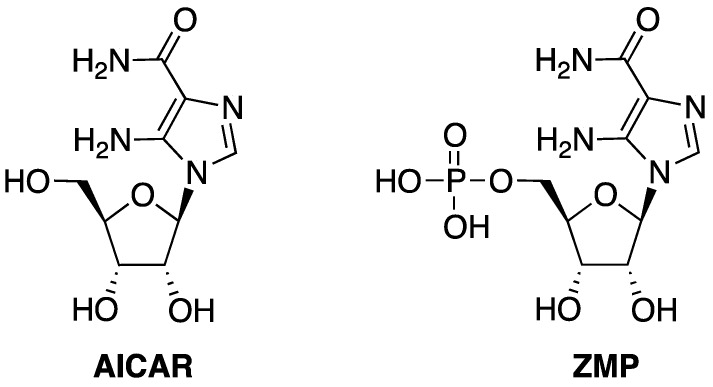
Structures of AICAR and ZMP.

New synthetic methodologies have been developed to prepare novel nucleoside and nucleotide analogues for enlarging the collection of potential antitumoral and antiviral drugs and for improving the pharmacological and pharmacokinetic properties of the existing ones [1,2,3,4,5,6,7,8,9,10]. Particular attention has been paid to imidazole nucleosides and nucleotides considering that their precursors, 5-aminoimidazole-4-carboxamide riboside (acadesine or AICAR) and 5-aminoimidazole-4-carboxamide ribotide (ZMP, Figure 1), are involved in the activation of AMP-activated protein kinase (AMPK) [11,12]. In the cells AICAR is phosphorylated to ZMP that is a mimic of AMP. The direct binding of ZMP to an allosteric site of AMPK causes a cascade of important metabolic events, including inhibition of the basal and insulin-stimulated glucose uptake, lipogenesis and glucose oxidation [13]. In the treatment of ischemia, AICAR has also been shown to be cardioprotective [14], preventing the endoplasmic reticulum stress, the inhibition of the sodium/hydrogen exchanger and thus the rapid rise of intracellular sodium, which normally occurs during the reperfusion [15].

The AMPK pathway is also implicated in the regulation of cell proliferation and activation by AICAR could result in pro-apoptotic effects [16,17]. Moreover, it was established that mutations affecting the Ca^2+^ releasing channel RYR1 are associated with a broad spectrum of human disorders, including malignant hyperthermia, central core disease and core-rod myopathy [18]. By using a mouse model of malignant hyperthermia having mutation in the RYR1 gene, Lanner *et al.* have recently demonstrated that AICAR can inhibit Ca^2+^ leakage through RYR1 by a mechanism independent from AMPK activation, thus preventing heat-induced sudden death in the mutated mouse [18]. Considering that AICAR has a short half-life in cells, does not cross efficiently the blood-brain barrier, is poorly adsorbed by the gastrointestinal tract, increases uric acid production and the lactic acidosis [15], the development of novel AICAR derivatives/analogues endowed with an improved pharmacological profile is an appealing goal in the field of medicinal chemistry.

In recent years we have focused our attention on the preparation of new base- and sugar-modified nucleosides and nucleotides [4,10,19,20,21,22,23,24] with the aim to enlarge the pool of new potential antimetabolites and to obtain new probes to better understand how certain metabolic pathways work. It is well known that the introduction of fluorine in a potential drug can dramatically change its chemical behaviour, influencing its pharmacokinetic and pharmacodynamics properties; furthermore, increased metabolic stability and a more efficient permeation of biological membranes are a consequence, respectively, of the strength of the C-F bond and of the higher hydrophobicity of fluorine respect to hydrogen [25,26,27].

**Scheme 1:**
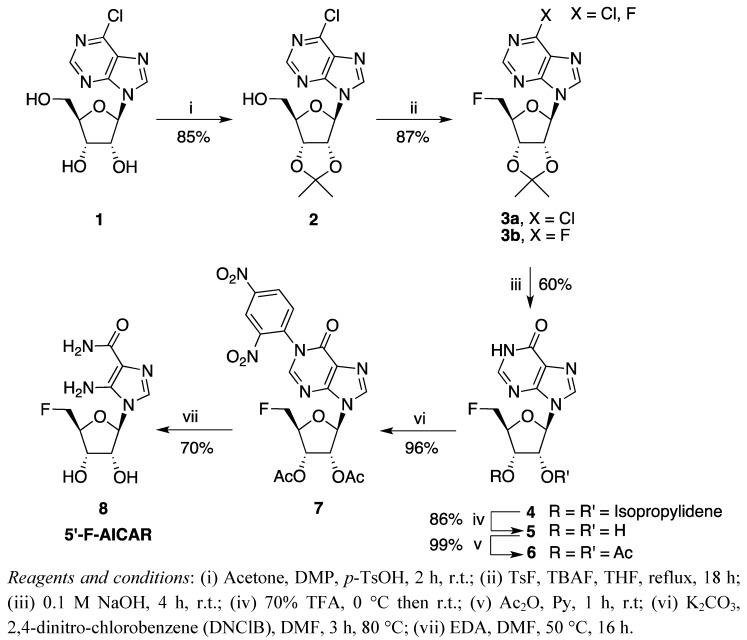
Preparation of compound 5′-F-AICAR **8**.

In the last years several novel synthetic methodologies have been proposed for the selective introduction of fluorine at the 2′-, 3′- or 5′-position of a nucleoside [28,29]; in some cases these substitutions were revealed to be so important that novel anti-tumor and anti-viral drugs have been obtained [28]. In this paper we report on the synthesis of the novel analogue 5′-fluoro-5′-deoxy-acadesine (5′-F-AICAR, **8**, Scheme 1) in which the 5′-hydroxyl group is replaced by a fluorine atom. The latter is an isopolar mimic of the hydroxyl group, but prevents the kinase-mediated 5′-phosphorylation by cellular kinases. We hypothesized that this new derivative, which can be considered the most similar and non-phosphorylable AICAR analogue, could be a useful tool to explore the wide and not completely understood physiological roles of AICAR and ZMP. Futhermore, 5′-F-AICAR may be also considered as a mimic of 5′-fluoro-5′-deoxyadenosine (5′-FDA) that has been employed as a tool for studying the Actinomycete bacterium *Strepromyces cattleya* [30]. Interestingly, some 5′-FDA derivatives were revealed to be inhibitors of the enzyme *S*-adenosyl-l-homocysteine hydrolase (an enzyme involved in the replication of some viruses) thanks to their chemical inertness toward the phosphorylation [30,31]. Hence, it would not be surprising if the novel compound 5′-F-AICAR could also work as an antiviral agent.

## 2. Results and Discussion

At first, we tried the selective insertion of the fluorine atom at the 5′-position of AICAR starting from its 2′,3′-*O*-isopropilydene derivative, using common fluorinating agents, such as: (**a**) DAST, (**b**) deoxofluor, (**c**) the Olah reagent [28] and (**d**) the tosyl fluoride (TsF)/tetrabutylammonium fluoride system (TBAF) [30]. Unfortunately, all these procedures failed, resulting in the recovery of only the unreacted starting material (reagent **d**) or decomposition of the nucleoside (reagents **a**–**c**). These results thus prompted us to explore an alternative synthetic route. We hypothesized that the construction of the 5-aminoimidazole-4-carboxamide moiety could be performed starting from an easily fluorinable purinic substrate, from which AICAR could be obtained through the degradation of the purine ring. In this context, we have recently reported efficient synthetic strategies to obtain AICAR, ZMP and their 4-*N*-alkyl derivatives, through the purine ring degradation of suitable inosine or 5′-phosphate inosine precursors, respectively, after the introduction of a suitable strong electron-withdrawing group (2,4-dinitrophenyl, DNP) at the N1 position of the base moiety [20,21,22,24]. 

Accordingly, for the preparation of 5′-F-AICAR (**8**) we used as starting material commercially available 6-chloronebularine (**1**, Scheme 1), which was readily transformed into its 2′,3′-*O*-isopropylidene derivative **2** [32]. Then, the 5′-OH group of **2** was replaced by a 5′-F in a one-pot tosylation/fluorination sequence, treating **2** with the TsF/TBAF reagent system, according to the Schimizu’s procedure [30]. It should be noted that the presence of an electron-withdrawing atom, such as chlorine, at the C6 position is necessary to prevent the intramolecular nucleophilic attack of N3 on the first-formed C5′-OTs, that would led to the unwanted cyclic side product containing the N3-C5′ bond [29]. As previously observed by Ashton *et al.* [30], we noted (TLC monitoring) that the process led not only to the formation of the expected fluorinated product **3a**, but also to the formation of the bis-fluorinated product **3b** (Scheme 1, X = F), as a consequence of the substitution in **2** of the C6 chlorine atom by fluoride. This was in no way detrimental to the continuation of the synthetic plan since both **3a** and **3b** could be used in the next step. Thus, the **3a**/**3b** mixture was converted into the corresponding inosine derivative **4 ** by reaction with a 0.1 M NaOH solution, affording **4** in a 60% yield. In particular, **3a** and **3b** were revealed to be reactive to the same extent toward the hydroxyl ion, and the displacement of chlorine and fluorine atoms at C6 could both be monitored by TLC.

Importantly, the success of this reaction was strongly dependent upon the concentration of the base employed. In fact, concentrations of NaOH higher than 0.1 M caused the decomposition of the nucleosidic material leading to the formation of several side-products, one of which was identified as 2′,3′-*O*-isopropylideneinosine.

Then, removal of the isopropylidene group in **4** with 70% trifluoroacetic acid (TFA) gave inosine derivative **5** [33], which was acetylated with Ac_2_O in pyridine under standard conditions to give the 2′,3′-di-*O*-acetylated product **6** quantitatively. The change of the 2′,3′-protecting group was necessary since the deprotection of 2′,3′-*O*-isopropylidene group in the final step led to a partial decomposition of the 5′-fluoro-acadesine **8**. Next, introduction in **6** of the strong DNP electron-withdrawing group at the N1 position was carried out by treatment with K_2_CO_3_ and 2,4-dinitrochlorobenzene (DNClB) to give **7** [34,35]. This process served to activate the C2 position of the purine, rendering it susceptible to nucleophilic attack by amines, thus leading to the cleavage of the N3-C2 bond. In particular, we have previously demonstrated that when a 1,ω-diaminoalkane is employed, the fate of the open intermediate depends on the length of the alkyl chain separating the two amino groups. If the diamine comprises two or three methylene groups, AICAR is formed as the sole product [22].

Eventually, when compound **7** was left into contact with a solution of ethylendiamine (EDA) in DMF, 5′-F-AICAR **8** was obtained by purine-ring opening and concomitant deacetylation of the hydroxyl groups at the 2′-3′ of the ribose moiety. During this reaction no loss of the fluorine at the 5′C position was observed. Compound **8** was obtained in 70% yield starting from **7** and an overall 25% yield from **1**. Its structure was confirmed by 1D/2D-NMR evidence and by high-resolution mass spectrometry (HRMS).

## 3. Experimental

### General

All the reagents were obtained from commercial sources (Sigma-Aldrich, city, state abbrev if USA, country) and were used without further purification. ^1^H-, ^13^C-, and ^19^F-NMR spectra were acquired on a Varian Mercury Plus 400 MHz and on a Varian Unit Inova 500 MHz in CD_3_OD. Chemical shifts are reported in parts per million (δ) relative to the residual solvent signals: CD_2_HOD 3.31 for ^1^H-NMR; CD_2_HOD 49.0 for ^13^C-NMR. ^1^H-NMR chemical shifts were assigned by 2D NMR experiments. Trifluorotoluene was used as external standard for ^19^F-NMR experiments. The abbreviations s, bs, d, dd, ddd and m stand for singlet, broad singlet, doublet, doublet of doublets, doublet doublet of doublets and multiplet, respectively. UV spectra were recorded on a Jasco V-530 UV spectrophotometer. High-resolution MS spectra were recorded on a Bruker APEX II FT-ICR mass spectrometer using electrospray ionization (ESI) technique in positive mode. IR spectra were recorded on a Jasco FT-IR 430 spectrophotometer. Column chromatography was performed by using silica gel 60 (70-230 mesh ASTM, Merck). Analytical TLC analyses were performed using F254 silica gel plates (0.2 mm thick, Merck). TLC spots were detected under UV light (254 nm).

*2′,3′-O-Isopropylidene-6-chloronebularine* (**2**). Compound **2** (102 mg) was obtained by reaction of **1** (100 mg, 0.37 mmol) according to the procedure by Kappler *et al.* [32]. Spectroscopic data and yields were in agreement with those reported by the authors. 

*5′-Fluoro-5′-deoxy-2′,3′-O-isopropylidene-6-chloronebularine* (**3a**) and 5′-fluoro-5′-deoxy-2′,3′-O-isopropylidene-6-fluoro-nebularine (**3b**). Compound **2** (102 mg, 0.31 mmol) was reacted according to the procedure by Ashton *et al.* [30]. Spectroscopic data and yields were in agreement with those reported by the authors. A mixture (86 mg) of **3a** (Rf = 0.48, hexane/AcOEt, 1:1) and **3b** (Rf = 0.42, hexane/AcOEt, 1:1) were obtained; HRESIM: *m/z* 351.0641 (**3a**), 335.0938 (**3b**) ([M+Na]^+^ C_13_H_14_ClFN_4_NaO_3_, requires 351.0636 for **3a**; [M+Na]^+^, C_13_H_14_F_2_N_4_NaO_3_, requires 335.0932 for **3b**).

*5′-Fluoro-5′-deoxy-2′,3′-O-isopropylideneinosine* (**4**). To a solution of **3a** and **3b** (86 mg, 0.27 mmol) in dioxane (5 mL) was a added 0.1 M NaOH solution (5 mL). The mixture was stirred at room temperature for 4 h (TLC monitoring: CHCl_3_/MeOH, 9:1, Rf = 0.29) and then neutralized with Dowex-50 resin (H^+^ form), previously washed with H_2_O, MeOH and then dried under vacuum. The mixture was filtered through a glass column [10 mm diameter, 100 mm length, with fused-in sintered glass-disc of porosity 3 (bore of plug: 2.5 mm)] and the resin was washed several times with MeOH and then the solvents were removed under reduced pressure. The crude was applied on a silica gel column eluted with increasing amounts of MeOH in CHCl_3_ (from 0 to 10%) to afford pure **4** (60%, 50 mg, 0.16 mmol) as an amorphous white solid. Spectroscopic data of **4** were in agreement with those reported by Spitale *et al.* [33].

*5′-Fluoro-5′-deoxyinosine* (**5**). Compound **4** (50 mg, 0.16 mmol) was reacted according to the procedure by Spitale *et al.* [33] to give 38 mg of **5**. Spectroscopic data and yields were in agreement with those reported by the authors. 

*5′-Fluoro-5′-deoxy-2′,3′-di-O-acetylinosine* (**6**). Compound **5** (38 mg, 0.14 mmol) was dissolved in a solution of Ac_2_O in pyridine (4:6, v/v, 2.0 mL) and the mixture was stirred at room temperature for 1 h (TLC monitoring: CHCl_3_/MeOH, 9:1). The solvents were removed under reduced pressure to afford compound **6** that was used for the next reaction step without further purification. Foam (99%, 49 mg, 0.14 mmol); ^1^H-NMR (400 MHz, CD_3_OD) δ 8.28 (bs, 1H, H-2), 7.87 (s, 1H, H-8), 6.27 (d, *J* = 5.1 Hz, 1H, H-1′), 5.86–5.81 (m, 1H, H-2′), 5.70–5.64 (m, 1H, H-3′), 4.84–4.63 (m, 2H, 2 × H-5′_a,b_), 4.53–4.40 (m, 1H, H-4′), 2.14 (s, 3H, CH_3_), 2.05 (s, 3H, CH_3_); ^13^C-NMR (100 MHz, CDCl_3_) δ 171.3, 171.0, 158.8, 149.8, 147.9, 140.5, 125.7, 87.9, 83.2 (d, *J*_CF_ = 170.8 Hz), 82.7 (d, *J*_CF_ = 18.1 Hz), 74.8, 71.2, 20.4, 20.2; ^19^F (400 MHz) δ (126.5–126.9) (m); *m*/*z* 377.0877 (HRESIMS) ([M+Na]^+^, C_14_H_15_FN_4_NaO_6_ requires 377.0873). IR (neat)*ν*_max_ 3054, 1751, 1698, 1376, 1242, 1214, 1099 cm^−1^. UV (MeOH) *λ*_max_ 250 nm.

*5′-Fluoro-5′-deoxy-2′,3′-di-O-acetyl-1-(2,4-dinitrophenyl)inosine* (**7**). A mixture of **6** (49 mg, 0.14 mmol), DNClB (169 mg, 0.56 mmol), and K_2_CO_3_ (77 mg, 0.56 mmol) was suspended in anhydrous DMF (3.0 mL) and stirred at 80 **°**C for 3 h. The reaction was monitored by TLC (CHCl_3_/MeOH, 95:5). After cooling, the mixture was filtered and the solid was washed with CHCl_3_. The filtrates and washings, collected and evaporated to dryness, were applied on a silica gel column eluted with increasing amounts of MeOH in CHCl_3_(from 0 to 5%) to give pure **7**, consisting of a 1:1 mixture of atropisomers at the *N*(1)-phenyl bond. Pale yellow amorphous solid (96%, 71 mg, 0.13 mmol); ^1^H-NMR (400 MHz, CD_3_OD) δ 9.03 (d, *J* = 2.3 Hz, 2H, 2 × H-3 DNP), 8.74 (dd, *J* = 8.6, 2.4 Hz, 2H, 2 × H-5 DNP), 8.49 (bs, 2H, 2 × H-2), 8.28 (bs, 2H, 2 × H-8), 8.01 (d, *J* = 8.6 Hz, 2H, 2 x H-6 DNP), 6.33 (t, *J* = 4.7 Hz, 2H, 2 × H-1′), 5.92–5.87 (m, 2H, 2 × H-2′), 5.74–5.68 (m, 2H, 2 × H-3′), 4.84–4.66 (m, 4H, 2 × H-5′_a,b_), 4.55–4.42 (m, 2H, 2 × H-4′_a,b_), 2.15 (bs, 6H, CH_3_), 2.09 (bs, 6H, CH_3_); ^13^C-NMR (100 MHz, CDCl_3_) δ 171.3, 171.1, 157.0, 149.7, 149.1, 148.4, 147.4, 141.4, 136.4, 133.4, 130.2, 124.9, 122.0, 88.1, 83.1 (d, *J*_CF_ = 171.1 Hz), 82.9 (d, *J*_CF_ = 17.8 Hz), 74.9, 71.3, 20.3, 20.2; ^19^F (400 MHz) δ (127.6–126.1) (m); *m*/*z* 543.0891 (HRESIMS) ([M+Na]^+^, C_20_H_17_FN_6_NaO_10_, requires 543.0888). UV (MeOH) *λ*_max_ 243 nm.

*5′-Fluoro-5′-deoxyacadesine (5′-F-AICAR,* **8**). Compound **7** (71 mg, 0.13 mmol) was dissolved in DMF (1.0 mL) and then EDA (0.17 mL, 2.6 mmol) was added. The mixture was stirred at 50 °C for 16 h (TLC monitoring: CHCl_3_/MeOH, 7:3) and then the solvents were removed under reduced pressure. The crude was applied on a silica gel column eluted with increasing amounts of MeOH in CHCl_3_ (from 0 to 20%) to afford pure **8**. Amorphous white solid (70%, 26 mg, 0.091 mmol); ^1^H-NMR (500 MHz, CD_3_OD) δ 7.32 (s, 1H, H-2), 5.58 (d, *J* = 5.7 Hz, 1H, H-1′), 4.71 (ddd, *J* = 47.5, 10.8, 2.3 Hz, 1H, H-5′_a_), 4.61 (ddd, *J* = 47.5, 10.8, 2.1 Hz, 1H, H-5′_b_); 4.36–4.32 (m, 1H, H-2′), 4.28–4.24 (m, 1H, H-3′), 4.23–4.13 (m, 1H, H-4′); ^13^C-NMR (100 MHz, CDCl_3_) δ 164.1, 145.2, 129.7, 113.6, 89.6, 84.9 (d, *J*_CF_ = 18.4 Hz), 83.8 (d, *J*_CF_ = 168.2 Hz), 75.1, 71.1; ^19^F (400 MHz) δ (100.9–101.3) (m); *m*/*z* 283.0822 (HRESIMS) ([M+Na]^+^, C_9_H_13_FN_4_NaO_4_, requires 283.0819 ). IR (neat)*ν*_max_ 3320, 1642, 1556, 1056 cm^−1^. UV (MeOH) *λ*_max_ 264 nm.

## 4. Conclusions

In this paper we have reported a facile synthetic approach for the preparation of the novel compound 5′-fluoro-5′-deoxyacadesine (5′-F-AICAR) starting from commercially available 6-chloro-nebularine (**1**). This product may be considered a novel non-phosphorylable AICAR derivative, whose biological activity has to be explored, and that could be a useful tool to understand the metabolic pathways involving AICAR and ZMP. The described synthetic procedure may be extended to the preparation of novel fluorinated base modified AICAR.
